# Diethyl 5-acetamido-3-methyl­thio­phene-2,4-dicarboxyl­ate

**DOI:** 10.1107/S1600536810045629

**Published:** 2010-11-13

**Authors:** Asma Mukhtar, M. Nawaz Tahir, Misbahul Ain Khan, Muhammad Naeem Khan

**Affiliations:** a Institute of Chemistry, University of the Punjab, Lahore, Pakistan; b Department of Physics, University of Sargodha, Sargodha, Pakistan; c Applied Chemistry Research Center, PCSIR Laboratories Complex, Lahore 54600, Pakistan

## Abstract

The title compound, C_13_H_17_NO_5_S, is approximately planar (r.m.s. deviation for the non-H atoms = 0.055 Å). Its conformation is stabilized by N—H⋯O and C—H⋯O hydrogen bonds, which both generate *S*(6) rings. The crystal packing only features van der Waals contacts.

## Related literature

For a related crystal structure and background, see: Mukhtar *et al.* (2010 ▶). For graph-set notation, see: Bernstein *et al.* (1995 ▶).
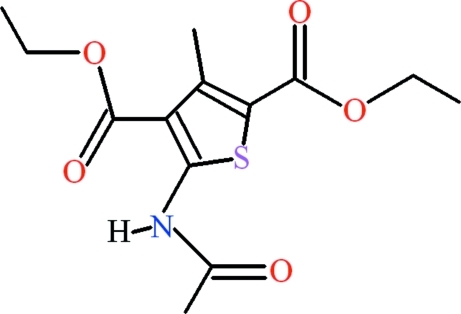


## Experimental

### 

#### Crystal data

C_13_H_17_NO_5_S
*M* *_r_* = 299.34
Monoclinic, 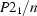

*a* = 15.933 (3) Å
*b* = 4.6028 (6) Å
*c* = 20.152 (3) Å
β = 106.005 (7)°
*V* = 1420.6 (4) Å^3^
*Z* = 4
Mo *K*α radiation
μ = 0.25 mm^−1^
*T* = 296 K
0.25 × 0.10 × 0.08 mm

#### Data collection

Bruker Kappa APEXII CCD diffractometer
Absorption correction: multi-scan (*SADABS*; Bruker, 2005 ▶) *T*_min_ = 0.972, *T*_max_ = 0.983
10222 measured reflections
2518 independent reflections
1311 reflections with *I* > 2σ(*I*)
*R*_int_ = 0.094

#### Refinement

*R*[*F* ^2^ > 2σ(*F* ^2^)] = 0.065
*wR*(*F* ^2^) = 0.172
*S* = 1.02
2518 reflections
186 parameters
H-atom parameters constrained
Δρ_max_ = 0.25 e Å^−3^
Δρ_min_ = −0.23 e Å^−3^

### 

Data collection: *APEX2* (Bruker, 2009 ▶); cell refinement: *SAINT* (Bruker, 2009 ▶); data reduction: *SAINT*; program(s) used to solve structure: *SHELXS97* (Sheldrick, 2008 ▶); program(s) used to refine structure: *SHELXL97* (Sheldrick, 2008 ▶); molecular graphics: *ORTEP-3 for Windows* (Farrugia, 1997 ▶) and *PLATON* (Spek, 2009 ▶); software used to prepare material for publication: *WinGX* (Farrugia, 1999 ▶) and *PLATON*.

## Supplementary Material

Crystal structure: contains datablocks global, I. DOI: 10.1107/S1600536810045629/hb5730sup1.cif

Structure factors: contains datablocks I. DOI: 10.1107/S1600536810045629/hb5730Isup2.hkl

Additional supplementary materials:  crystallographic information; 3D view; checkCIF report

## Figures and Tables

**Table 1:** Hydrogen-bond geometry (Å, °)

*D*—H⋯*A*	*D*—H	H⋯*A*	*D*⋯*A*	*D*—H⋯*A*
N1—H1⋯O2	0.86	1.99	2.652 (5)	133
C10—H10*B*⋯O4	0.96	2.24	2.995 (6)	135

**Fig. 1.**
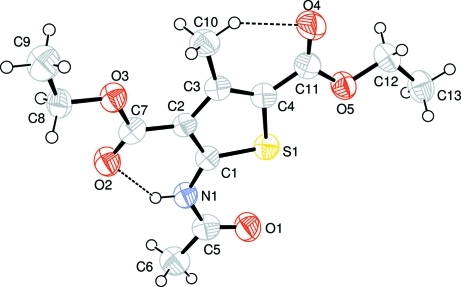
View of the title compound with displacement ellipsoids drawn at the 50% probability level. H-atoms are shown as small spheres of arbitrary radii. The dotted lines represents the intramolecular H-bondings.

## supplementary crystallographic information

### Comment

We have reported the synthesis and crystal structure of (II) *i.e.*, Ethyl 2-benzamido-4,5,6,7-tetrahydro-1-benzothiophene-3-carboxylate (Mukhtar *et al.*, 2010). The title compound (I, Fig. 1) is being reported here in continuation to synthesize various thiophene derivatives.

The title compound essentially consists of monomers. In (I), the methylthiophene group A (C1—C4/S1/C10), acetamide group B (N1/C5/C6/O1), ethylester groups C (O2/C7/O3/C8/C9) and D (O4/C11/O5/C12/C13) are planar with r. m. s. deviation of 0.0049, 0.0033, 0.0224 and 0.0082 Å, respectively. The dihedral angle between A/B, A/C, A/D, B/C, B/D and C/D is 5.55 (29), 7.30 (32), 6.24 (25), 10.40 (36), 10.51 (29) and 12.08 (32)°, respectively. In the title compound two S(6) ring motifs (Bernstein *et al.*, 1995) are formed due to intramolecular H-bondings of C—H···O and N—H···O types (Table 1, Fig. 1). There does not exist any appreciable π interaction.

### Experimental

A mixture of (0.3 g, 1 mmol) diethyl 2-amino-4-methylthiophene- 3,5-dicarboxylate, dissolved in chloroform and 0.1 ml of acetyl chloride was heated at 330 K for 10 h. The solvent of resultant product was removed and the residue was recrystallized from ethanol to give orange needles of the title compound. m.p. 394 K; yield: 0.25 g; 85%.

### Refinement

The H-atoms were positioned geometrically (N—H = 0.86, C–H = 0.96–0.97 Å) and refined as riding with *U*_iso_(H) = *xU*_eq_(C, N), where *x* = 1.5 for methyl and *x* = 1.2 for all other H-atoms.

### Crystal data

**Table:**

C_13_H_17_NO_5_S	*F*(000) = 632
*M**_r_* = 299.34	*D*_x_ = 1.400 Mg m^−^^3^
Monoclinic, *P*2_1_/*n*	Mo *K*α radiation, λ = 0.71073 Å
Hall symbol: -P 2yn	Cell parameters from 1311 reflections
*a* = 15.933 (3) Å	θ = 2.1–25.1°
*b* = 4.6028 (6) Å	µ = 0.25 mm^−^^1^
*c* = 20.152 (3) Å	*T* = 296 K
β = 106.005 (7)°	Needle, orange
*V* = 1420.6 (4) Å^3^	0.25 × 0.10 × 0.08 mm
*Z* = 4	

### Data collection

**Table:**

Bruker Kappa APEXII CCD diffractometer	2518 independent reflections
Radiation source: fine-focus sealed tube	1311 reflections with *I* > 2σ(*I*)
graphite	*R*_int_ = 0.094
Detector resolution: 8.20 pixels mm^-1^	θ_max_ = 25.1°, θ_min_ = 2.1°
ω scans	*h* = −18→18
Absorption correction: multi-scan (*SADABS*; Bruker, 2005)	*k* = −5→5
*T*_min_ = 0.972, *T*_max_ = 0.983	*l* = −20→24
10222 measured reflections	

### Refinement

**Table:**

Refinement on *F*^2^	Secondary atom site location: difference Fourier map
Least-squares matrix: full	Hydrogen site location: inferred from neighbouring sites
*R*[*F*^2^ > 2σ(*F*^2^)] = 0.065	H-atom parameters constrained
*wR*(*F*^2^) = 0.172	*w* = 1/[σ^2^(*F*_o_^2^) + (0.0583*P*)^2^ + 0.3244*P*] where *P* = (*F*_o_^2^ + 2*F*_c_^2^)/3
*S* = 1.02	(Δ/σ)_max_ < 0.001
2518 reflections	Δρ_max_ = 0.25 e Å^−^^3^
186 parameters	Δρ_min_ = −0.22 e Å^−^^3^
0 restraints	Extinction correction: *SHELXL97* (Sheldrick, 2008), Fc^*^=kFc[1+0.001xFc^2^λ^3^/sin(2θ)]^-1/4^
Primary atom site location: structure-invariant direct methods	Extinction coefficient: 0.011 (2)

### Special details

**Table:**

Geometry. Bond distances, angles *etc*. have been calculated using the rounded fractional coordinates. All su's are estimated from the variances of the (full) variance-covariance matrix. The cell e.s.d.'s are taken into account in the estimation of distances, angles and torsion angles
Refinement. Refinement of *F*^2^ against ALL reflections. The weighted *R*-factor *wR* and goodness of fit *S* are based on *F*^2^, conventional *R*-factors *R* are based on *F*, with *F* set to zero for negative *F*^2^. The threshold expression of *F*^2^ > σ(*F*^2^) is used only for calculating *R*-factors(gt) *etc*. and is not relevant to the choice of reflections for refinement. *R*-factors based on *F*^2^ are statistically about twice as large as those based on *F*, and *R*- factors based on ALL data will be even larger.

### Fractional atomic coordinates and isotropic or equivalent isotropic displacement parameters (Å^2^)

**Table:**

	*x*	*y*	*z*	*U*_iso_*/*U*_eq_	
S1	0.31949 (8)	0.0384 (2)	0.49921 (6)	0.0506 (5)	
O1	0.2674 (2)	0.0997 (7)	0.35864 (17)	0.0745 (17)	
O2	0.1065 (2)	0.7124 (7)	0.48056 (18)	0.0619 (12)	
O3	0.1245 (2)	0.6438 (6)	0.59347 (17)	0.0568 (12)	
O4	0.4142 (3)	−0.1121 (7)	0.6963 (2)	0.0805 (17)	
O5	0.4407 (2)	−0.2748 (6)	0.59982 (16)	0.0583 (11)	
N1	0.1981 (2)	0.3879 (7)	0.4170 (2)	0.0505 (16)	
C1	0.2371 (3)	0.2886 (8)	0.4826 (3)	0.0431 (16)	
C2	0.2152 (3)	0.3811 (9)	0.5411 (2)	0.0441 (17)	
C3	0.2680 (3)	0.2433 (9)	0.6015 (2)	0.0448 (16)	
C4	0.3277 (3)	0.0560 (9)	0.5869 (2)	0.0470 (17)	
C5	0.2156 (3)	0.2958 (10)	0.3575 (3)	0.058 (2)	
C6	0.1661 (3)	0.4472 (11)	0.2930 (3)	0.072 (2)	
C7	0.1450 (3)	0.5918 (9)	0.5342 (3)	0.0504 (19)	
C8	0.0519 (3)	0.8434 (10)	0.5883 (3)	0.0568 (19)	
C9	0.0353 (4)	0.8550 (11)	0.6579 (3)	0.081 (2)	
C10	0.2625 (3)	0.2958 (10)	0.6742 (2)	0.0622 (19)	
C11	0.3963 (3)	−0.1159 (10)	0.6338 (3)	0.0559 (19)	
C12	0.5140 (3)	−0.4391 (10)	0.6418 (3)	0.067 (2)	
C13	0.5539 (3)	−0.6035 (10)	0.5932 (3)	0.0692 (19)	
H1	0.15902	0.52060	0.41285	0.0604*	
H6A	0.18642	0.38122	0.25496	0.1074*	
H6B	0.17496	0.65304	0.29852	0.1074*	
H6C	0.10496	0.40458	0.28401	0.1074*	
H8A	0.00031	0.77509	0.55396	0.0687*	
H8B	0.06655	1.03516	0.57495	0.0687*	
H9A	0.08548	0.93427	0.69089	0.1219*	
H9B	0.02430	0.66237	0.67177	0.1219*	
H9C	−0.01457	0.97565	0.65561	0.1219*	
H10A	0.27447	0.49656	0.68603	0.0934*	
H10B	0.30462	0.17622	0.70576	0.0934*	
H10C	0.20497	0.24802	0.67713	0.0934*	
H12A	0.49428	−0.57346	0.67146	0.0802*	
H12B	0.55670	−0.30905	0.67064	0.0802*	
H13A	0.57324	−0.46835	0.56419	0.1038*	
H13B	0.51108	−0.73156	0.56500	0.1038*	
H13C	0.60277	−0.71498	0.61936	0.1038*	

### Atomic displacement parameters (Å^2^)

**Table:**

	*U*^11^	*U*^22^	*U*^33^	*U*^12^	*U*^13^	*U*^23^
S1	0.0497 (8)	0.0563 (7)	0.0508 (9)	0.0004 (6)	0.0222 (6)	−0.0001 (6)
O1	0.081 (3)	0.088 (3)	0.057 (3)	0.026 (2)	0.023 (2)	−0.0013 (19)
O2	0.063 (2)	0.076 (2)	0.052 (2)	0.0152 (18)	0.025 (2)	0.0064 (18)
O3	0.056 (2)	0.068 (2)	0.054 (2)	0.0086 (16)	0.0279 (18)	0.0036 (16)
O4	0.095 (3)	0.094 (3)	0.050 (3)	0.029 (2)	0.016 (2)	0.005 (2)
O5	0.055 (2)	0.0638 (19)	0.057 (2)	0.0108 (16)	0.0172 (18)	0.0064 (16)
N1	0.054 (3)	0.059 (2)	0.044 (3)	0.0083 (18)	0.023 (2)	0.003 (2)
C1	0.041 (3)	0.047 (2)	0.046 (3)	−0.005 (2)	0.020 (2)	0.002 (2)
C2	0.042 (3)	0.049 (3)	0.045 (3)	−0.004 (2)	0.018 (2)	−0.005 (2)
C3	0.041 (3)	0.050 (2)	0.047 (3)	−0.009 (2)	0.018 (3)	0.000 (2)
C4	0.046 (3)	0.054 (3)	0.046 (3)	−0.006 (2)	0.021 (2)	0.002 (2)
C5	0.061 (4)	0.067 (3)	0.052 (4)	−0.007 (3)	0.025 (3)	−0.001 (3)
C6	0.078 (4)	0.094 (4)	0.050 (4)	0.005 (3)	0.029 (3)	0.005 (3)
C7	0.051 (3)	0.051 (3)	0.056 (4)	−0.012 (2)	0.026 (3)	−0.004 (3)
C8	0.046 (3)	0.065 (3)	0.063 (4)	0.009 (2)	0.021 (3)	−0.002 (3)
C9	0.075 (4)	0.114 (4)	0.070 (4)	0.016 (3)	0.046 (3)	0.001 (3)
C10	0.065 (4)	0.078 (3)	0.047 (3)	0.005 (3)	0.021 (3)	0.000 (3)
C11	0.053 (3)	0.057 (3)	0.058 (4)	−0.002 (2)	0.016 (3)	0.000 (3)
C12	0.058 (4)	0.070 (3)	0.068 (4)	0.010 (3)	0.010 (3)	0.010 (3)
C13	0.054 (3)	0.076 (3)	0.083 (4)	0.016 (3)	0.028 (3)	0.008 (3)

### Geometric parameters (Å, °)

**Table:**

S1—C1	1.709 (5)	C8—C9	1.498 (8)
S1—C4	1.738 (4)	C12—C13	1.510 (7)
O1—C5	1.219 (6)	C6—H6A	0.9600
O2—C7	1.220 (6)	C6—H6B	0.9600
O3—C7	1.344 (6)	C6—H6C	0.9600
O3—C8	1.458 (6)	C8—H8A	0.9700
O4—C11	1.212 (7)	C8—H8B	0.9700
O5—C11	1.331 (6)	C9—H9A	0.9600
O5—C12	1.452 (6)	C9—H9B	0.9600
N1—C1	1.375 (7)	C9—H9C	0.9600
N1—C5	1.371 (7)	C10—H10A	0.9600
N1—H1	0.8600	C10—H10B	0.9600
C1—C2	1.386 (7)	C10—H10C	0.9600
C2—C3	1.424 (6)	C12—H12A	0.9700
C2—C7	1.458 (7)	C12—H12B	0.9700
C3—C10	1.511 (6)	C13—H13A	0.9600
C3—C4	1.374 (6)	C13—H13B	0.9600
C4—C11	1.464 (7)	C13—H13C	0.9600
C5—C6	1.494 (8)		
			
C1—S1—C4	90.4 (3)	H6A—C6—H6B	109.00
C7—O3—C8	115.5 (4)	H6A—C6—H6C	109.00
C11—O5—C12	116.3 (4)	H6B—C6—H6C	109.00
C1—N1—C5	126.3 (4)	O3—C8—H8A	110.00
C1—N1—H1	117.00	O3—C8—H8B	110.00
C5—N1—H1	117.00	C9—C8—H8A	110.00
S1—C1—N1	122.1 (4)	C9—C8—H8B	110.00
N1—C1—C2	124.3 (4)	H8A—C8—H8B	109.00
S1—C1—C2	113.6 (4)	C8—C9—H9A	109.00
C1—C2—C7	119.3 (4)	C8—C9—H9B	109.00
C1—C2—C3	111.3 (4)	C8—C9—H9C	109.00
C3—C2—C7	129.4 (4)	H9A—C9—H9B	109.00
C2—C3—C10	125.4 (4)	H9A—C9—H9C	109.00
C2—C3—C4	112.2 (4)	H9B—C9—H9C	109.00
C4—C3—C10	122.4 (4)	C3—C10—H10A	110.00
C3—C4—C11	129.7 (4)	C3—C10—H10B	109.00
S1—C4—C11	117.7 (4)	C3—C10—H10C	110.00
S1—C4—C3	112.6 (3)	H10A—C10—H10B	109.00
O1—C5—N1	120.8 (5)	H10A—C10—H10C	109.00
O1—C5—C6	123.7 (5)	H10B—C10—H10C	109.00
N1—C5—C6	115.5 (4)	O5—C12—H12A	110.00
O2—C7—O3	121.4 (4)	O5—C12—H12B	110.00
O3—C7—C2	113.7 (4)	C13—C12—H12A	110.00
O2—C7—C2	124.9 (5)	C13—C12—H12B	110.00
O3—C8—C9	107.3 (4)	H12A—C12—H12B	108.00
O4—C11—O5	122.4 (5)	C12—C13—H13A	109.00
O4—C11—C4	125.6 (5)	C12—C13—H13B	110.00
O5—C11—C4	111.9 (4)	C12—C13—H13C	110.00
O5—C12—C13	107.3 (4)	H13A—C13—H13B	109.00
C5—C6—H6A	110.00	H13A—C13—H13C	109.00
C5—C6—H6B	110.00	H13B—C13—H13C	109.00
C5—C6—H6C	109.00		
			
C4—S1—C1—N1	178.4 (4)	N1—C1—C2—C7	1.7 (7)
C4—S1—C1—C2	−0.8 (4)	C1—C2—C3—C4	0.6 (6)
C1—S1—C4—C3	1.1 (4)	C1—C2—C3—C10	179.3 (4)
C1—S1—C4—C11	−177.0 (4)	C7—C2—C3—C4	179.9 (5)
C8—O3—C7—O2	2.2 (6)	C7—C2—C3—C10	−1.4 (8)
C8—O3—C7—C2	−177.3 (4)	C1—C2—C7—O2	−5.3 (7)
C7—O3—C8—C9	175.9 (4)	C1—C2—C7—O3	174.1 (4)
C12—O5—C11—O4	−1.7 (7)	C3—C2—C7—O2	175.3 (5)
C12—O5—C11—C4	176.1 (4)	C3—C2—C7—O3	−5.2 (7)
C11—O5—C12—C13	179.5 (4)	C2—C3—C4—S1	−1.1 (5)
C5—N1—C1—S1	3.2 (6)	C2—C3—C4—C11	176.7 (5)
C5—N1—C1—C2	−177.7 (4)	C10—C3—C4—S1	−179.9 (3)
C1—N1—C5—O1	3.2 (7)	C10—C3—C4—C11	−2.1 (8)
C1—N1—C5—C6	−177.9 (4)	S1—C4—C11—O4	174.8 (4)
S1—C1—C2—C3	0.3 (5)	S1—C4—C11—O5	−2.9 (5)
S1—C1—C2—C7	−179.2 (3)	C3—C4—C11—O4	−2.9 (8)
N1—C1—C2—C3	−178.9 (4)	C3—C4—C11—O5	179.4 (4)

### Hydrogen-bond geometry (Å, °)

**Table:**

*D*—H···*A*	*D*—H	H···*A*	*D*···*A*	*D*—H···*A*
N1—H1···O2	0.86	1.99	2.652 (5)	133
C10—H10B···O4	0.96	2.24	2.995 (6)	135
